# Cardiac Arrest and Resuscitation in Pregnancy: A Case Report

**DOI:** 10.7759/cureus.36771

**Published:** 2023-03-27

**Authors:** Preeti Singh, Anuja Bhalerao

**Affiliations:** 1 Department of Obstetrics and Gynaecology, NKP Salve Institute of Medical Sciences and Research Centre, Nagpur, Maharashtra, IND

**Keywords:** advanced cardiovascular life support, cardiopulmonary resuscitation, maternal cardiac arrest, manual left uterine displacement, resuscitative hysterotomy

## Abstract

Maternal mortality is a major public health issue globally. The exact incidence of maternal cardiac arrest (CA) is uncertain in developing countries like India as there is a paucity of reliable maternal registries. CA in pregnancy is rare and requires a multidisciplinary team well versed in the cascade of steps during cardiopulmonary resuscitation (CPR) in managing such patients. Here we report a case of a 29-year-old primigravida at 29 weeks antenatal woman in cardiac arrest in which CPR with advanced care life support was initiated and resuscitative hysterotomy was performed within 4 minutes of no return of spontaneous circulation, which helped in the revival of the patient. It serves as an important basis for maternal health and the delivery of healthy neonates, as seen in our case. Because of this, it is crucial to have a comprehensive understanding of pregnancy physiology as well as basic and advanced cardiac life support techniques with a focus on CA in pregnancy. Frequent simulation learning and training on the treatment of CA in pregnancy should therefore be encouraged.

## Introduction

Cardiac arrest (CA), which is uncommon in pregnancy, is one of the most difficult clinical situations complicating about 1 in 30,000 pregnancies [1]. The primary distinction is that it involves two lives, the mother and the fetus. As a result, in order to prevent and treat CA during pregnancy as effectively as possible, carers must have a good grasp of maternal mortality [2]. Amniotic fluid embolism, pulmonary thromboembolic events, sepsis, hemorrhagic shock, and problems from anesthesia are just various reasons for CA in pregnancy. Additionally, congenital cardiac conditions, anaphylaxis, and trauma are uncommon causes [3].

Mothers are treated with cardiopulmonary resuscitation (CPR) in the same way as other patients, with a few minor modifications due to pregnancy-related alterations [4]. To provide proper care for both mother and the neonate, the departments of obstetrics and neonatology should be included at a very early stage [1]. Increased oxygen demand, reduced chest compliance, increased ventilation, an ineffective gastroesophageal (cardiac) sphincter, increased intragastric pressure, an increased risk of regurgitation, and decreased functional residual capacity are few of the physiological changes that late pregnancy brings about, correspondingly affecting CPR [5].

Once a respiratory or cardiac arrest has been found, it is essential to act quickly, with the patient properly positioned and basic life support started immediately. This must continue while venous access is obtained, any evident causes (such as hypovolaemia) are treated, and the required medicines, staff, and devices are gathered [5].

After CA has occurred, gaps in knowledge and inadequate resuscitation techniques may play a significant role in demonstrating poor results [6-8]. Considering these issues, new statistics indicate that after maternal CA, the survival probability till the patient gets discharged can be as high as 58.9% [9], higher than the majority of populations, which further supports the need for effective training and planning for such situations regardless of their rarity. Therefore, this case report is aimed to highlight the rare case of CA in pregnancy followed by resuscitative hysterotomy.

## Case presentation

**Figure 1:**
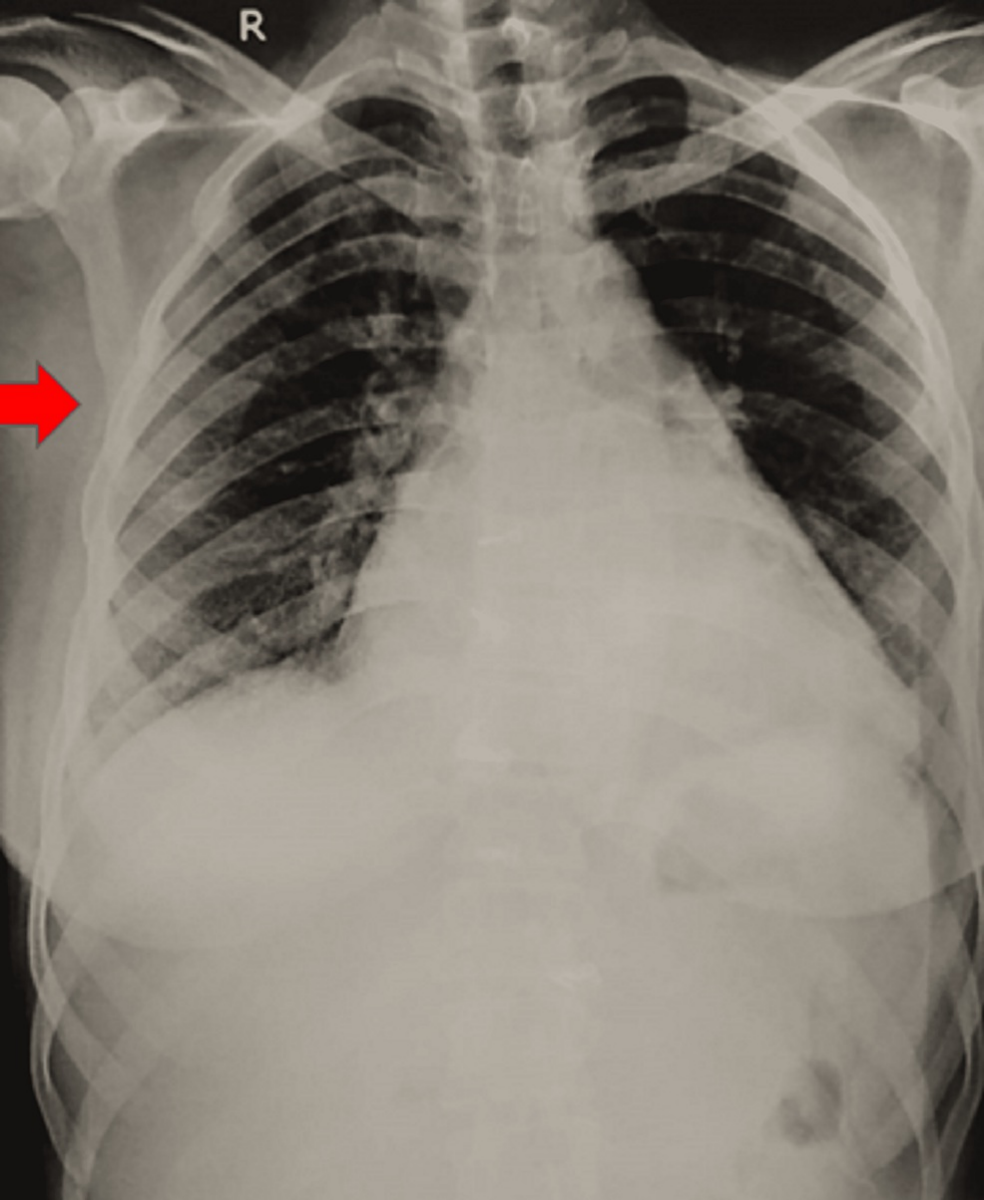
Red arrow highlighting pulmonary oedema.

A 29-year-old primigravida at 29 weeks of gestation presented to the casualty department with chief complaints of difficulty in breathing for 1 hour along with severe respiratory distress. On examination, the vital parameters of the patient demonstrated poor general condition, was febrile, with 140 beats/min of heart rate and 150/90 mmHg of blood pressure. Additionally, the respiratory rate was 40 breaths/minute and peripheral capillary oxygen saturation (SpO2) was 52% on room air. Respiratory system examination revealed extensive crepitations with pink frothy sputum. Further investigations on chest X-ray revealed sepsis with severe pulmonary arterial hypertension having pulmonary arterial pressure of 25 mmHg with pulmonary edema (Figure 1). Meanwhile, a 2-D echocardiography of the patient revealed rheumatic heart disease with mitral regurgitation. Injection Lasix (furosemide) 40 mg was given stat and the patient was shifted to the intensive care unit (ICU) under oxygen support of 1.5 litres.

In the ICU, the patient became drowsy and developed a decreased response to commands. Soon code blue was activated and immediate CPR was initiated by a team of anesthetists, obstetricians, and the nursing community according to the protocol of advanced cardiac life support (ACLS). The patient was intubated immediately, intravenous access was obtained and injection adrenaline was given. The patient was administered with 200 Joules direct current (DC) shock and after 4 minutes of no return of spontaneous circulation (ROSC). Resuscitative hysterotomy was performed, and after 8 minutes of delivery of the infant, there was a spontaneous return of maternal circulation. The baby was shifted to the neonatal ICU. The patient was shifted to the operating room for completion after the abdomen and the opened uterus were closed. An injection furosemide drip with a dose of 40 mg was started and electrolyte correction was given. Additionally, the patient was given injection meropenem 1 g and injection linezolid 600 mg and was kept on inotropes and a ventilator. Within a day repeated chest X-rays showed improvement in the pulmonary edema component. On day 2, the patient developed monomorphic ventricular tachycardia with a blood pressure of 70/ 30 mmHg. DC shock was given and CPR was commenced. As per the cardiologist's opinion, the patient was started on injection amiodarone drip 150 mg stat, followed by 1 mg/min drip. The patient was monitored by 12 hourly ECG and hourly BP charting. Eventually, the amiodarone drip was weaned off and relatives were counseled about the need for valve replacement surgery in the near future. The patient was continued with injection meropenem 1 g and injection linezolid 600 mg.

The patient was discharged on day 50 from the hospital. Later the patient underwent valve replacement surgery.

## Discussion

This case report describes favourable outcomes that were obtained with resuscitative hysterotomy following CA and concluded that positive and effective outcomes following maternal CA were observed, which requires a systematic approach and comprehensive information regarding anatomical and physiological modifications that occur during pregnancy. Although there is a growing emphasis on reducing maternal mortality in developing countries, it is still important for obstetricians to be prepared to manage CA during pregnancy for which rapid and accurate resuscitation with CA is essential for recovery and survival since it increases the likelihood that the foetus will survive, as observed in the present case [10].

With a fatality rate of 42% and survival rate of 15-20%, CA in pregnancy is unusual [1,3,10]. The same compression rate, depth, and hand placement should be used as with non-pregnant ones during resuscitation; however, the compressions should be conducted a little high in the chest since the gravid uterus elevates the diaphragm, thus moving the heart upward [3]. In order to allow for full recoil of the chest, chest compressions at a depth of at least 2 inches (5 cm) with minimal interruptions and a compression-ventilation ratio of 30:2 should be performed at a pace of at least 100 per minute in pregnant patients [2].

Applying cricoid pressure can avoid the aspiration of stomach contents because progesterone relaxes the gastroesophageal sphincters, making the patient more susceptible to it [2]. Early securing of the airway should be taken into consideration since significant chest rise with bag-mask ventilation might not be possible [2]. Above the level of the diaphragm, intravenous access should be established preferably and possible etiologies of CA should be explored. After 20 weeks of pregnancy, because of the gravid uterus causing aortocaval compression, the uterus should be moved off from the aorta, vena cava, and pelvic great vessels [3]. Manual left uterine displacement is advised as a technique for aortocaval decompression, which can be performed with one hand or with two hands as the use of a wedge or board to place the mother at a 25 to 30-degree left lateral tilt angle is no longer recommended due to the difficulty in achieving effective chest compressions in that position [11]. If ROSC cannot be achieved within 4 minutes after the start of CPR, resuscitative hysterotomy should be performed. As the removal of the foetus improves the mother's circulation by 20% to 25% during CPR, it leads to the enhancement of maternal survival. This directly improves neonatal outcomes and neurological damage due to hypoxia is avoided [2,3].

The airway, respiration, and circulation all undergo major alterations during pregnancy. Additionally, desaturation and increased oxygen demand are more likely when there is a decreased oxygen reserve during pregnancy [11,12]. Due to the laxity of lower oesophageal sphincters, there is also a need for reduced ventilation volumes and a higher probability of aspiration [11]. It is important to note that during defibrillation, foetal or uterine monitors should be removed as they cause loss of adequate cardiac shock dose.

Previously, a similar case was reported that was managed successfully with immediate emergency hysterotomy [13]. Timing and speed of resuscitative hysterotomy and stabilization of the mother determine the outcomes for the mother and the foetus; therefore, all emergency physicians should be well versed with the algorithm and should be trained in the procedure.

## Conclusions

The most severe pregnancy complication, CA, continues to harm both the woman and the foetus despite the best efforts of obstetricians to perform resuscitation. An important basis for maternal health and delivery of healthy neonates as seen in our case is resuscitative hysterotomy, for which obstetricians, anaesthesiologists, neonatologists, and nursing staff work in coordination in a multi-specialty team environment. Because of this, it is crucial to have a comprehensive understanding of pregnancy physiology as well as basic ACLS techniques with a focus on CA in pregnancy. Frequent simulation learning and training on the treatment of CA in pregnancy should therefore be encouraged.
